# Noninvasive assessment of skin barrier function: evaluating ceramide-based moisturizer using confocal Raman spectroscopy

**DOI:** 10.1117/1.JBO.30.S3.S34117

**Published:** 2025-12-31

**Authors:** Poongkulali Rajarahm, Renzhe Bi, Amalina Ebrahim Attia, Ruochong Zhang, Steven Tien Guan Thng, Sue Phay Ng, Linzhu Yuan, Malini Olivo

**Affiliations:** a Agency for Science Technology and Research, Translational Biophotonics Laboratory, A*STAR Skin Research Labs, Singapore; b National Skin Centre, Singapore; c Hyphens Pharma Pte Ltd, Singapore; d China-Singapore International Joint Research Institute, Guangzhou, China

**Keywords:** confocal Raman spectroscopy, atopic dermatitis, ceramide, handheld probe, dermatology

## Abstract

**Significance:**

Confocal Raman spectroscopy (CRS) is a noninvasive technique that enables detailed biochemical analysis of the skin, providing insights into its structural and molecular composition. Atopic dermatitis and hand eczema, both characterized by impaired skin barrier function, require effective monitoring tools to assess treatment efficacy.

**Aim:**

We aimed to quantitatively evaluate changes in skin physiological and biochemical parameters following the application of a ceramide-based moisturizer (test cream) compared with an aqueous moisturizer (control cream) in healthy volunteers and eczema patients.

**Approach:**

Skin physiological assessments and CRS measurements were performed to evaluate water content and ceramide levels in both superficial and deeper skin layers after application of the two moisturizers.

**Results:**

CRS revealed a significant increase in water content following test cream application, indicating improved skin hydration, whereas physiological measurements detected no statistically significant changes, underscoring the greater sensitivity of CRS. The test cream also significantly enhanced ceramide levels in both superficial and deeper skin layers, whereas the control cream increased ceramide levels only in the superficial stratum corneum (SC).

**Conclusions:**

These findings suggest that the test cream has a substantial positive impact on hand eczema, potentially improving both skin barrier function and biochemical properties.

## 1 Introduction

Raman spectroscopy, a technique based on the inelastic scattering of photons when molecules are excited, has emerged as a pivotal tool in biomedical applications such as DNA/RNA analysis, cell sorting, and disease diagnosis, owing to its noninvasive nature, exceptional specificity, and high sensitivity. Confocal Raman spectroscopy (CRS), with its depth profiling capabilities, has shown significant promise in dermatology by offering both structural and biochemical insights into skin.^1^^–^^3^ It enables rapid, noninvasive identification of crucial skin components such as ceramides, natural moisturizing factors (NMF), urea, and water content, which are essential for assessing skin barrier function and diagnosing skin disorders.^4^^,^^5^

Atopic dermatitis (AD), the most common form of eczema, is a chronic inflammatory skin disease characterized by intense itching, recurrent eczematous lesions, and a fluctuating course. AD affects individuals of all ages, with a lifetime prevalence of up to 20%, but it is significantly more common in children than in adults.^6^^,^^7^ Patients with AD often experience a higher prevalence of hand eczema, which manifests as erythema, vesicles, papules, scaling, fissures, hyperkeratosis, pruritus, and pain.^8^^,^^9^ Hand eczema, which has a lifetime prevalence of ∼15%,^10^^,^^11^ is frequently caused by chronic exposure to occupational allergens and irritants, leading to epithelial barrier dysfunction.^9^^,^^12^^,^^13^ This dysfunction can result in increased transepidermal water loss (TEWL) and alterations in the composition of stratum corneum (SC) proteins and lipids.^14^ Hand eczema not only causes significant socioeconomic consequences but also has a substantial negative impact on the quality of life of affected individuals.^9^

The SC, the outermost layer of the skin, plays a critical role in maintaining skin barrier function and homeostasis. In patients with AD, elevated skin surface pH and altered SC composition, including disrupted ceramide profiles, are characteristic features.^15^^–^^17^ These changes contribute to increased inflammation, compromised antimicrobial defense, and impaired hydration.^18^^,^^19^ Monitoring key skin parameters, such as ceramide concentration, skin pH, and TEWL, is essential for understanding and managing these conditions effectively.

Current CRS systems have demonstrated significant promise in noninvasive skin monitoring, offering capabilities for depth profiling and biochemical analysis. However, their bulky, benchtop configurations limit their usability for flexible, real-time assessments across various anatomical regions. Furthermore, dual-wavelength CRS systems often rely on sequential scanning, where measurements at the fingerprint region (450 to $1750\;{\mathrm{cm}}^{-1}$) are taken first, followed by separate scans at the high-wavenumber region (2800 to $3800\;{\mathrm{cm}}^{-1}$).^20^^,^^21^ This sequential approach increases acquisition times and introduces challenges such as potential depth mismatches caused by patient or motor movement. These limitations hinder the system’s practical application in dynamic clinical settings.^22^

To address these limitations, we have developed a novel fiber-based, simultaneous dual-wavelength CRS system with a handheld probe, allowing for seamless access to various body areas such as the face, arms, and legs.^22^^,^^23^ This portable system provides a faster, more practical, and flexible approach to skin analysis. In this study, we leveraged this advanced system to evaluate the effects of two moisturizers—a ceramide test cream and an aqueous control cream—on the skin physiology and biochemical composition of patients with and without hand eczema. Comprehensive measurements of ceramide concentration, water content, TEWL, and moisture levels were conducted to assess the efficacy of both creams. The innovative CRS device enabled highly efficient, real-time analysis of skin biochemical components, offering a state-of-the-art, noninvasive method for monitoring skin health and enhancing treatment strategies for AD and related conditions.^24^

## 2 Methods

### 2.1 Subjects

Participants were adults aged ≥21 years including 10 healthy volunteers (HV) with no pre-existing skin conditions and 10 eczema patients (EP) with mild eczema on both hands. Participants were required to have no topical products (other than investigational products) applied to their hands on the day of the study visit. Subjects who had any medical condition that, in the investigator’s judgment, made them inappropriate for study participation were excluded from the study. The study was approved by the National Healthcare Group (NHG) Domain Specific Review Board (DSRB), Singapore (Ref No. 2021/00869). All subjects provided written informed consent. Patient-informed consent was obtained in compliance with the institutional approvals. All methods were performed in accordance with the relevant guidelines and regulations.

### 2.2 Objectives

The objective was to quantify and evaluate changes in skin physiological (TEWL, skin surface moisture content) and biochemical parameters (CRS) before and 2 h after a single topical application of test cream versus control cream.

### 2.3 Moisturizer Application

A single topical application of a ceramide test cream—Ceradan Advanced Hand Balm (Hyphens Pharma, Singapore)—was administered (two fingertip units) to the palm of one hand of each subject, and a control cream—Basic Aquacream (ICM Pharma, Singapore)—applied (two fingertip units) to the palm of the other hand in an investigator-blinded manner. The test cream contains a buffering system and skin physiological lipids (ceramide, cholesterol, and free fatty acid) in a 3:1:1 ratio. Subjects were assessed for experience of stinging, irritation, or redness after the application of the moisturizers. Skin physiological and CRS measurements were conducted 2 h after moisturizer application (without washing).

### 2.4 Skin Physiological and CRS Measurements

Skin parameters were measured on the palms of both hands for each subject. TEWL was measured using a VapoMeter^®^ (Delfin Technologies, Kuopio, Finland), and skin surface moisture was measured using a MoistureMeter SC (Delfin Technologies, Kuopio, Finland). The biochemical measurements were extracted from our proprietary CRS system. Subjects were acclimatized for 15 to 30 min at an ambient temperature of 21°C to 25°C and room humidity of 60% to 76% prior to physiological measurements. Measurements were taken at one location on each palm. In addition, skin surface pH was measured using a skin pH meter (HORIBA, Kyoto, Japan). The results are presented in Fig. S1 in the Supplementary Material.

**Fig. 1:**
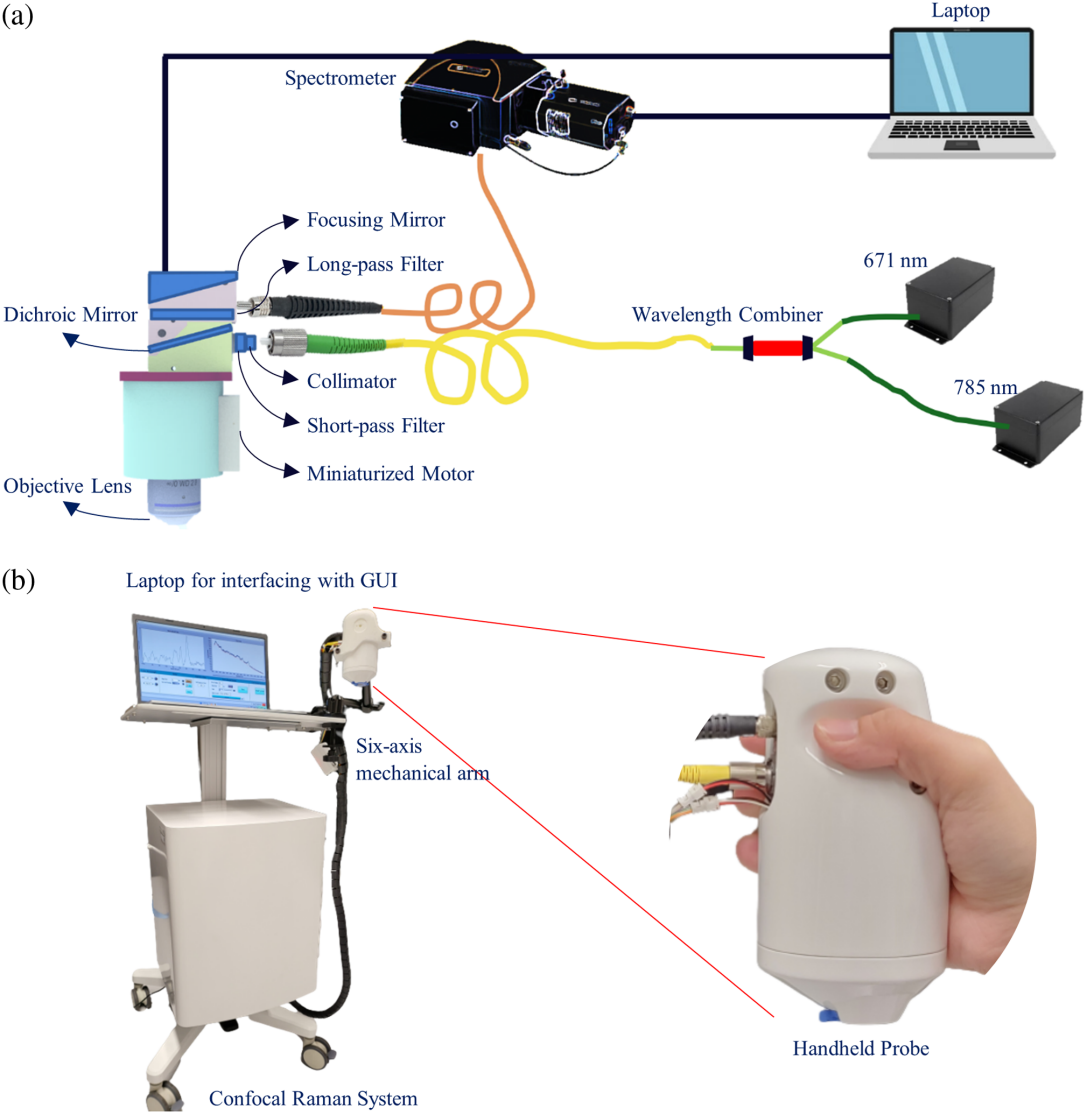
(a) Schematic of internal parts of dual-wavelength CRS system with a handheld probe. (b) Photograph of the third-generation prototype of the in-house built CRS system.

In this study, we utilized a portable, dual-wavelength excitation handheld CRS system that was developed in-house.^22^^,^^23^ This advanced system enables quasi-simultaneous spectral acquisition in both the fingerprint (FP, 450 to $1750\;{\mathrm{cm}}^{-1}$) and high-wavenumber (HW, 2800 to $3800\;{\mathrm{cm}}^{-1}$) regions. The skin surface was illuminated by 785 nm (IPS, 19.4 mW) and 671 nm (CrystaLaser, 4.2 mW) lasers to collect data from the FP and HW regions, respectively. The system utilizes a single-mode fiber for laser excitation and a multimode fiber for signal collection, both integrated into the probe. A microscope objective lens (NIR Apo 60×1.0  W, Nikon) is mounted on a custom miniaturized linear motor within the probe, enabling precise confocal depth profiling. The setup is interfaced with an optical spectrometer from Andor Technology, as illustrated in Fig. 1. To enable *in vivo* skin measurements, the system integrates a flexible handheld probe. Inside the probe, a miniaturized precision motor adjusts the objective lens position, allowing depth-resolved measurements across 25 discrete layers with a mechanical step size of 10  μm. Each spectrum is acquired with an integration time of 3 seconds per layer, with two spectra captured at each depth—one using the 785-nm excitation laser and the other with the 671-nm excitation laser. For patients with eczema (EP), Raman spectra were collected from both representative eczema lesions and non-lesional (control) areas.

### 2.5 Data Processing

**Fig. 2:**
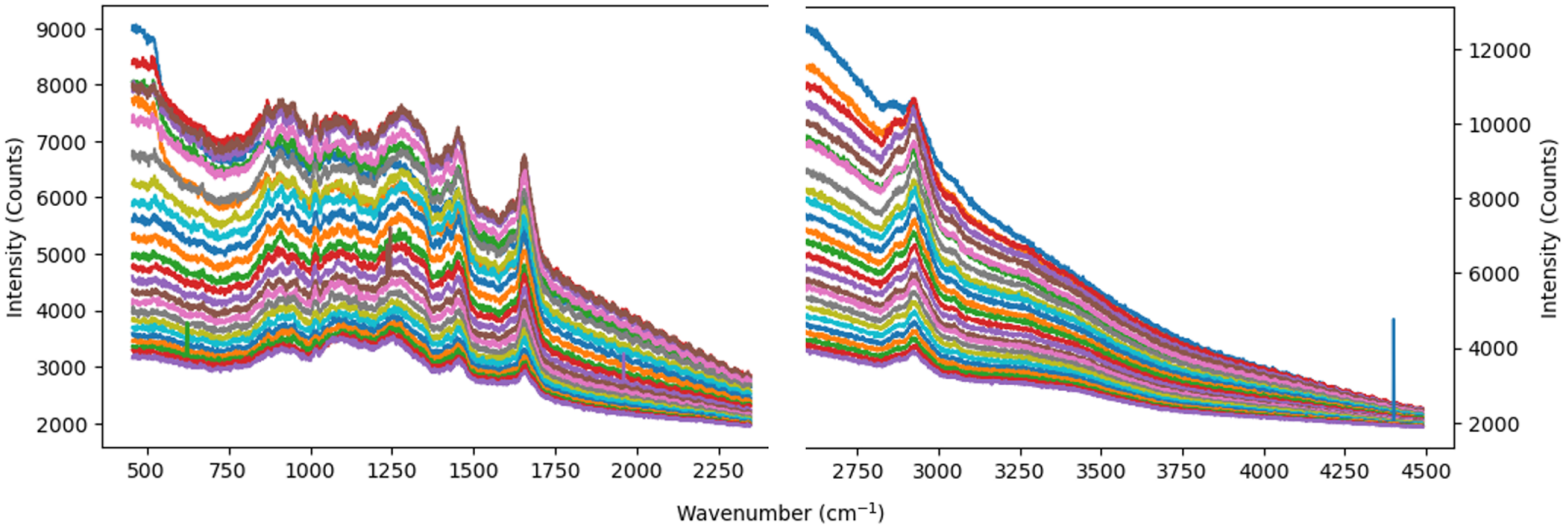
Raw Raman spectra of the FP and HW regions acquired at 25 distinct skin depths.

Before performing quantitative analysis, the raw spectra underwent a series of processing steps. Figure 2 shows the raw spectra of a subject acquired at 25 various depths in both the FP and HW regions. To this individual spectrum, a median filter of a window size of 3 is applied to remove spike artifacts caused by cosmic rays.^25^ This window size is smaller than the spectral resolution of the system ($∼13\;{\mathrm{cm}}^{-1}$), ensuring efficient noise removal while preserving genuine Raman peaks.

**Fig. 3:**
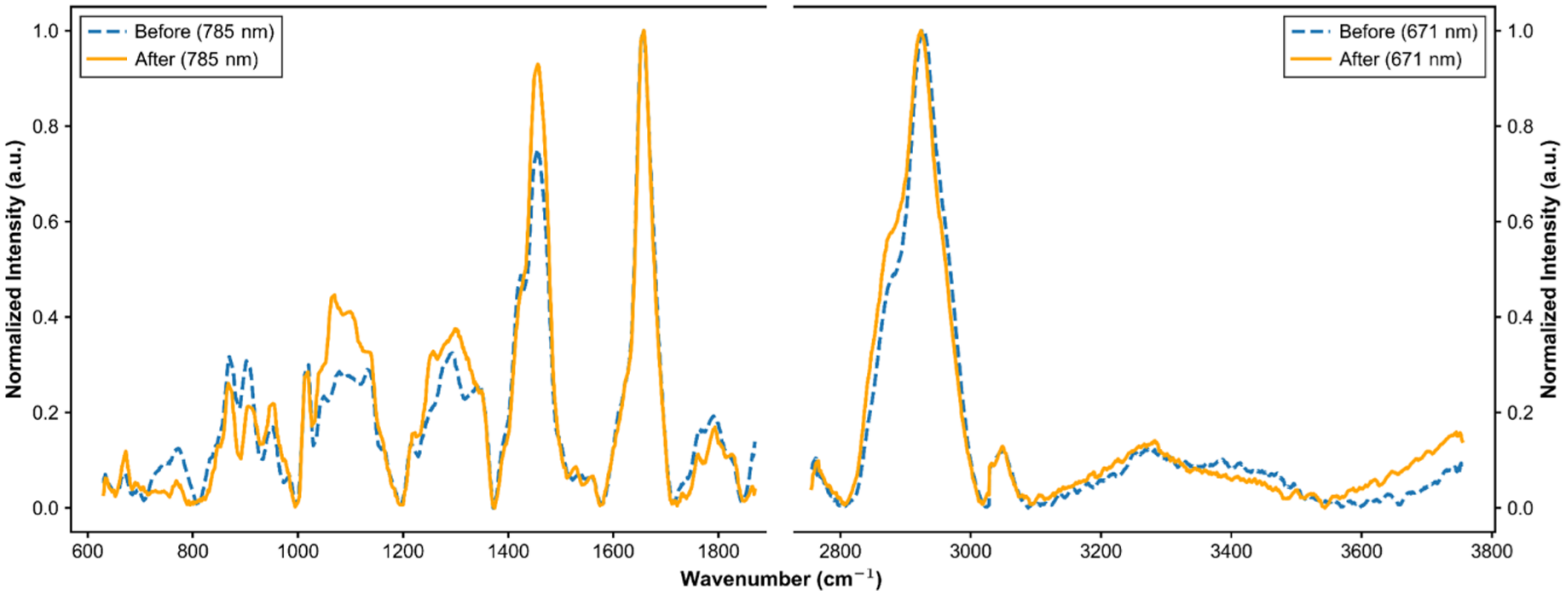
Spectra of the FP and HW regions after fluorescence removal and normalization. The blue plot shows the subject before, and the orange plot after, ceramide test cream application.

One of the major challenges in Raman measurements of endogenous tissue is the autofluorescence produced by intrinsic molecules or components naturally present within the tissue. This strong fluorescence background interferes with the detection of weaker Raman signals. Unlike Raman peaks, which are sharp and well-defined, fluorescence produces a broadband, continuous background that interferes with spectral analysis. To address this issue, polynomial fitting is commonly used to model the fluorescence baseline without interfering with the Raman peaks.^26^ Once the baseline is estimated, it is subtracted from the raw spectrum, leaving only the true Raman signals. This process, known as baseline correction, improves spectral clarity and enhances the accuracy of Raman measurements. The spectra are then smoothened to remove other unnecessary noises that cause a loss of significant information. Figure 3 shows the baseline corrected, smoothed and normalized spectra of the same subject before and after ceramide test cream application.

To extract component information, the FP and HW spectra are processed differently. For the FP region, the baseline-corrected spectrum was unmixed using non-negative least squares (NNLS) fitting in Python to obtain depth profiles of key SC components. The non-negative coefficients for each component at each depth were extracted by unmixing the spectrum against the Raman component library. This library was created by measuring the Raman spectra of each pure component and normalizing them to unit norm prior to the unmixing process. Given that keratin is the predominant component of the SC, it serves as a key marker for analysing skin surface characteristics.^27^ The skin surface was defined at the depth where the keratin amide I peak at $1655\;{\mathrm{cm}}^{-1}$ reaches its maximum intensity, and spectra from depths prior to this point were excluded from further processing.^28^ Key SC components, including keratin and ceramide, were extracted by unmixing the spectra in the FP region. Ceramide exhibits characteristic peaks at 888, 1060, 1127, 1294, and $1436\;{\mathrm{cm}}^{-1}$, associated with acyl chain vibrations. These characteristic peaks were used to assess compositional variations related to ceramide content.

In the HWN region, the water content is assessed by computing the values in the water region (3350 to $3550\;{\mathrm{cm}}^{-1}$) in the baseline-corrected spectrum. After processing each depth within the SC, the mean water and ceramide content values across all relevant layers are calculated. Statistical analysis is then performed to determine the p value and significance.

Quantitative data were summarized by mean, standard deviation (SD), number (n), and percentage. T tests were used to compare the means of treatment groups. A p value<0.05 was considered to be statistically significant. Data were displayed graphically by box and whisker plots.

## 3 Results

Characteristics of participants are shown in Table 1. The mean age of HV (n=10) and EP (n=10) was 37.1 and 35.2 years, respectively. The proportion of males was 20% and 70%, respectively.

**Table 1:** Demographic characteristics of the study population.

		Healthy volunteers (n=10)	Eczema patients (n=10)
Age (years)	Age range	29 to 47	28 to 44
	Mean age	37.1	35.2
Sex, n (%)	Male/female	2 (20)/8 (80)	7 (70)/3 (30)
Ethnic group, n (%)	Chinese	7 (70)	8 (80)
	Malay	3 (30)	2 (20)

**Fig. 4:**
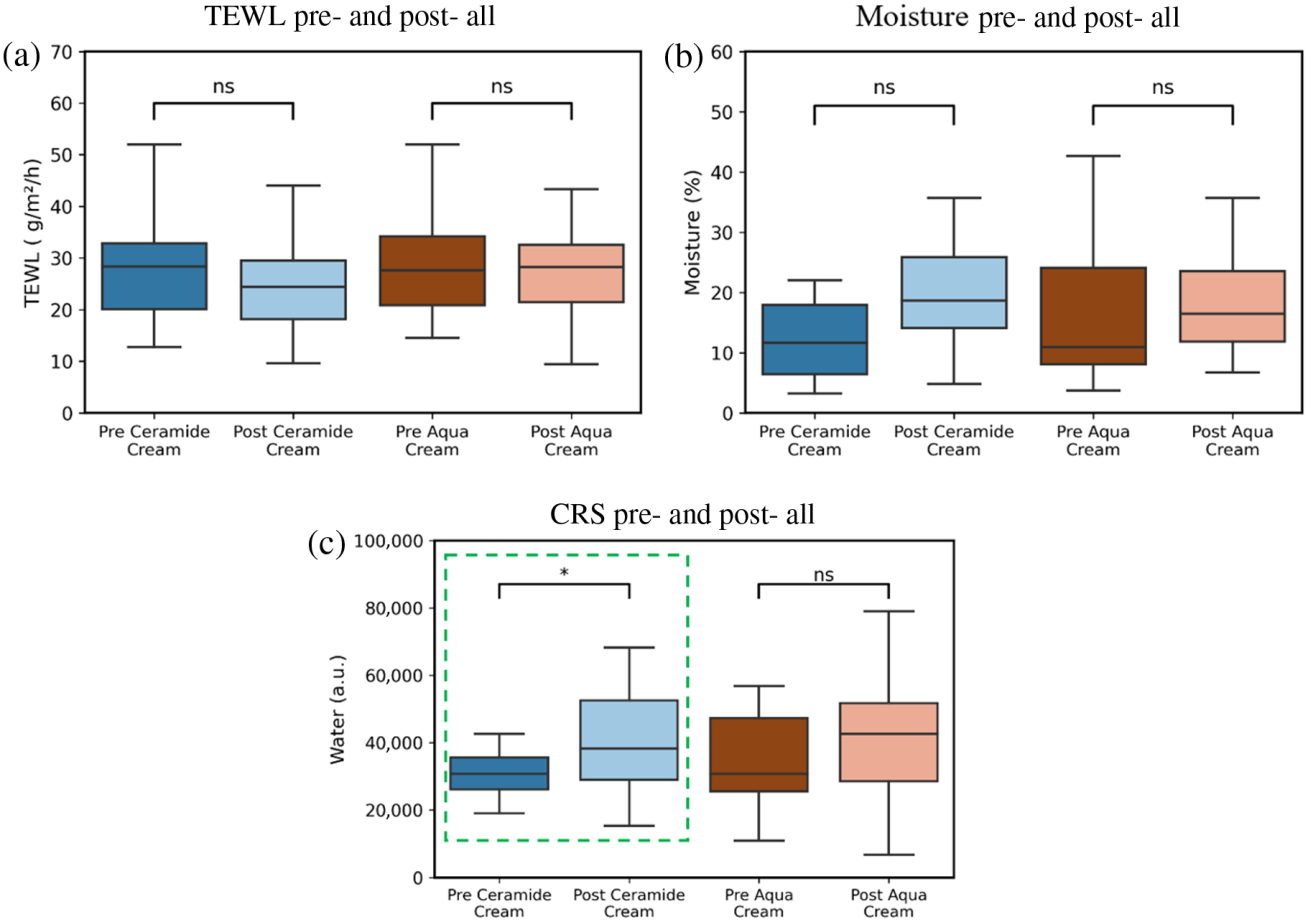
Box plots of physiological and CRS water measurements showing the difference between pre- and post-application of ceramide cream and aqueous cream (*p<0.05; ns: p>0.05). (a) TEWL, (b) moisture meter, and (c) box plots showing water content quantified from the O–H stretching band between 3350 and $3550\;{\mathrm{cm}}^{-1}$, extracted from HW spectra.

Elevated TEWL indicates barrier dysfunction, leading to increased water loss and dehydration. Because TEWL is inversely related to skin moisture content, its monitoring provides insight into barrier efficacy. A decrease in TEWL following hydration treatments suggests improved moisture retention and barrier function. At baseline (pre-cream application), no significant differences in TEWL, moisture content, or water ratio were observed between HV and EP. However, post-treatment analysis revealed promising trends. Although a decreasing trend in TEWL [Fig. 4(a)] and an increasing trend in moisture content [Fig. 4(b)] were observed on the test cream side, these changes were not statistically significant. By contrast, the water content measured using the CRS showed a significant increase post-treatment on the test cream side [Fig. 4(c)]. This suggests that CRS is a more reliable method for quantifying skin moisture and hydration compared with TEWL measurements.

**Fig. 5:**
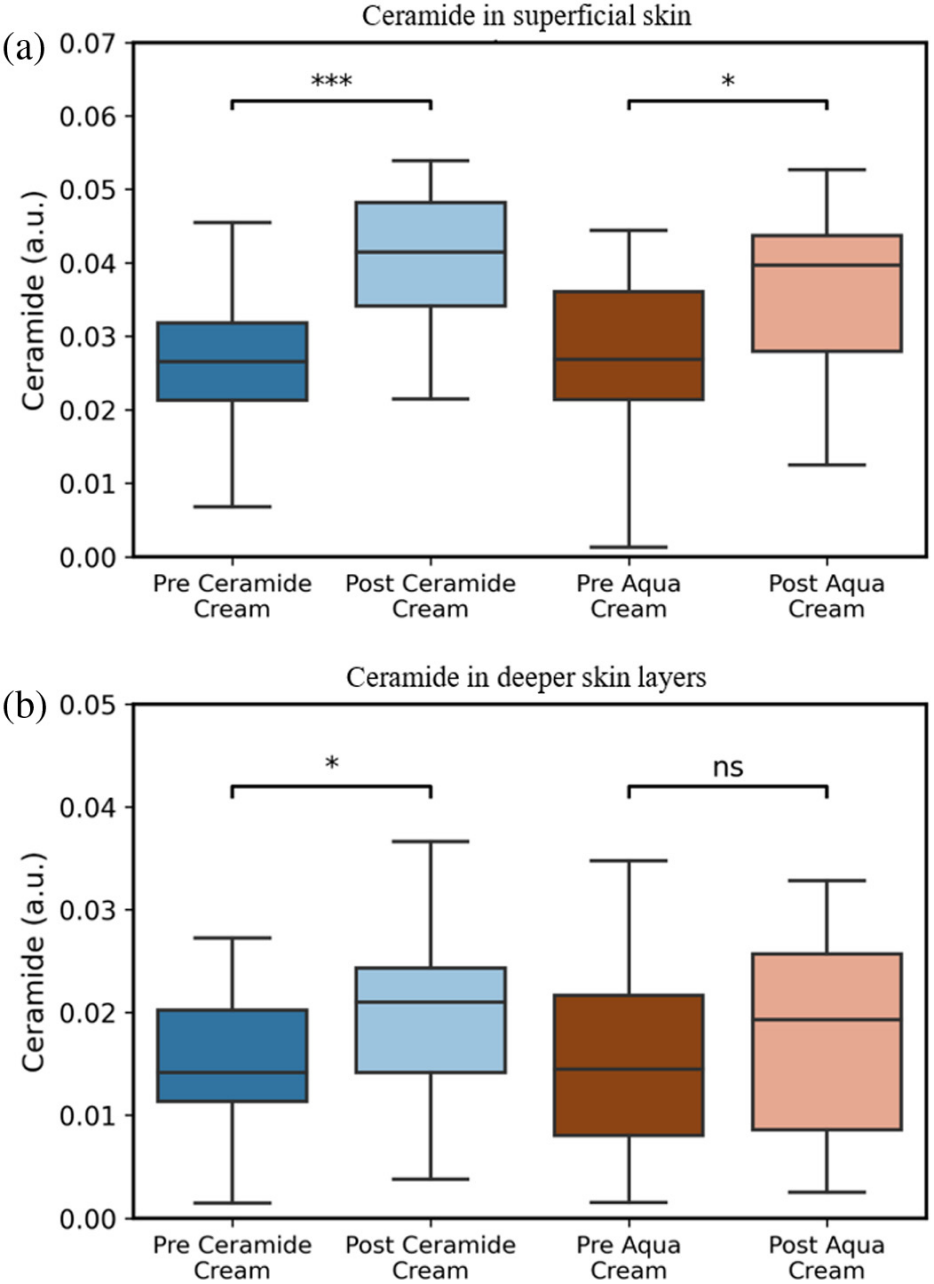
(a) Ceramide content quantified from characteristic peaks (888 to $1436\;{\mathrm{cm}}^{-1}$) in superficial skin layers of all subjects (n=20), pre- and post-application of the test cream or control cream. *p<0.05; ***p<0.001. (b) Ceramide content quantified from characteristic peaks (888 to $1436\;{\mathrm{cm}}^{-1}$) in deeper skin layers in all subjects (n=20) pre- and post-application of test cream or control cream. *p<0.05; n.s.: nonsignificant.

Because this study involved a ceramide-based test cream, ceramide levels were analyzed in greater detail using data acquired from CRS. The CRS-derived ceramide measurements were categorized into superficial SC layers (0 to 20  μm) and deeper SC layers (20 to 50  μm), and ceramide levels were extracted for both pre- and post-application of the test and control creams. Application of the test cream resulted in a significant increase in ceramide levels across both superficial and deeper skin layers as shown in Fig. 5. By contrast, the control cream led to a significant increase in ceramide levels only in the superficial layers [Fig. 5(a)], with no notable change observed in the deeper layers [Fig. 5(b)]. The test cream, however, produced a more pronounced and consistent increase in ceramide content across all layers.

**Fig. 6:**
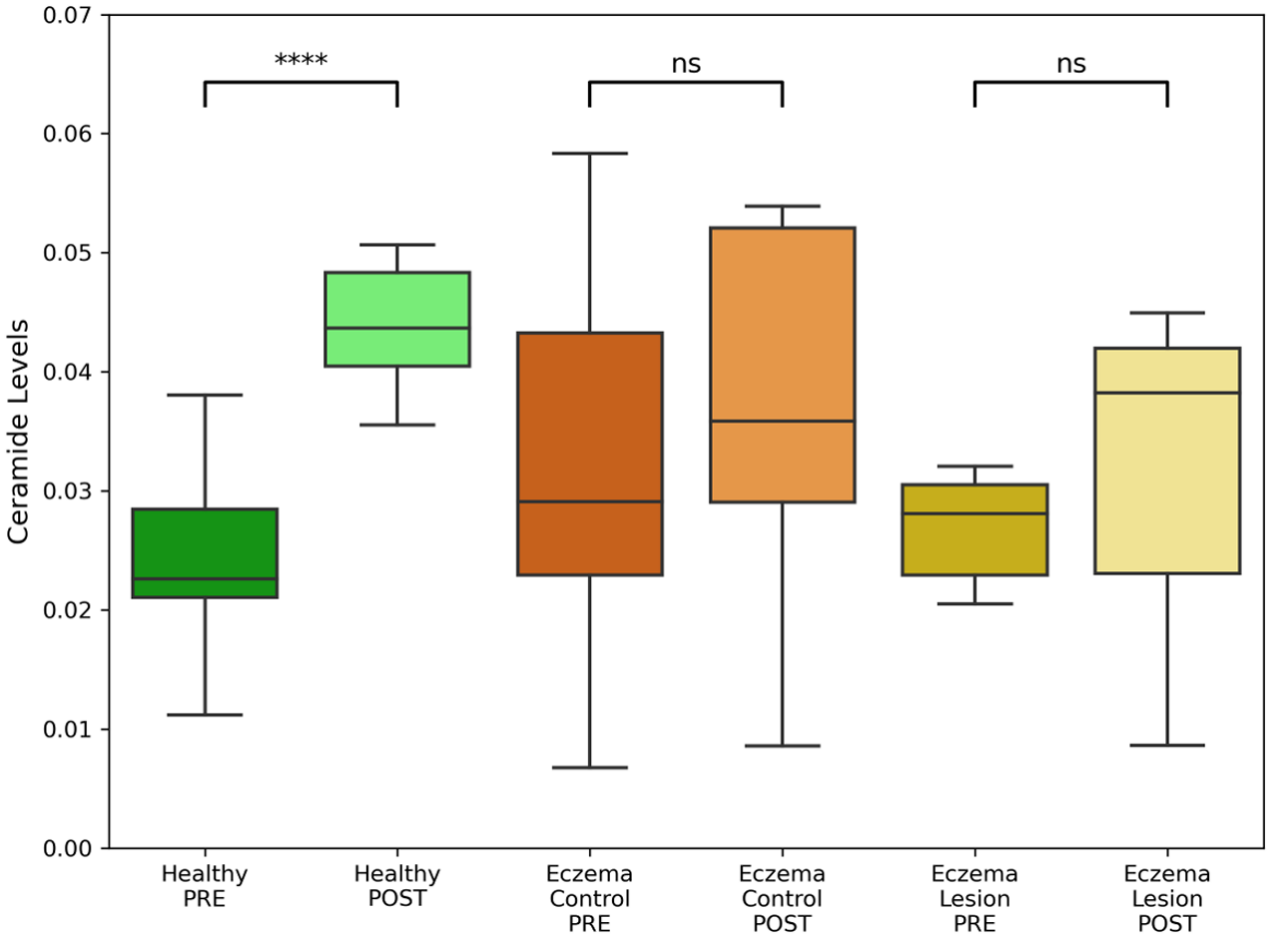
Ceramide content quantified from characteristic peaks (888 to $1436\;{\mathrm{cm}}^{-1}$) in superficial skin layers in healthy volunteers, eczema patients’ lesions or non-lesional (control) areas, pre- and post-application of test cream. ****p<0.0001; n.s.: nonsignificant.

After confirming that the test cream elevated ceramide levels across all layers of the SC, further analysis was conducted to evaluate the extent of its effects. Ceramide levels were categorized pre- and post-application across three distinct groups: healthy, eczema control, and eczema lesion. This analysis was performed exclusively on the test cream side to assess its efficacy in these groups. Although all three groups demonstrated an increasing trend in ceramide levels within the superficial layers, a statistically significant increase was observed solely in the healthy group, as illustrated in Fig. 6.

**Fig. 7:**
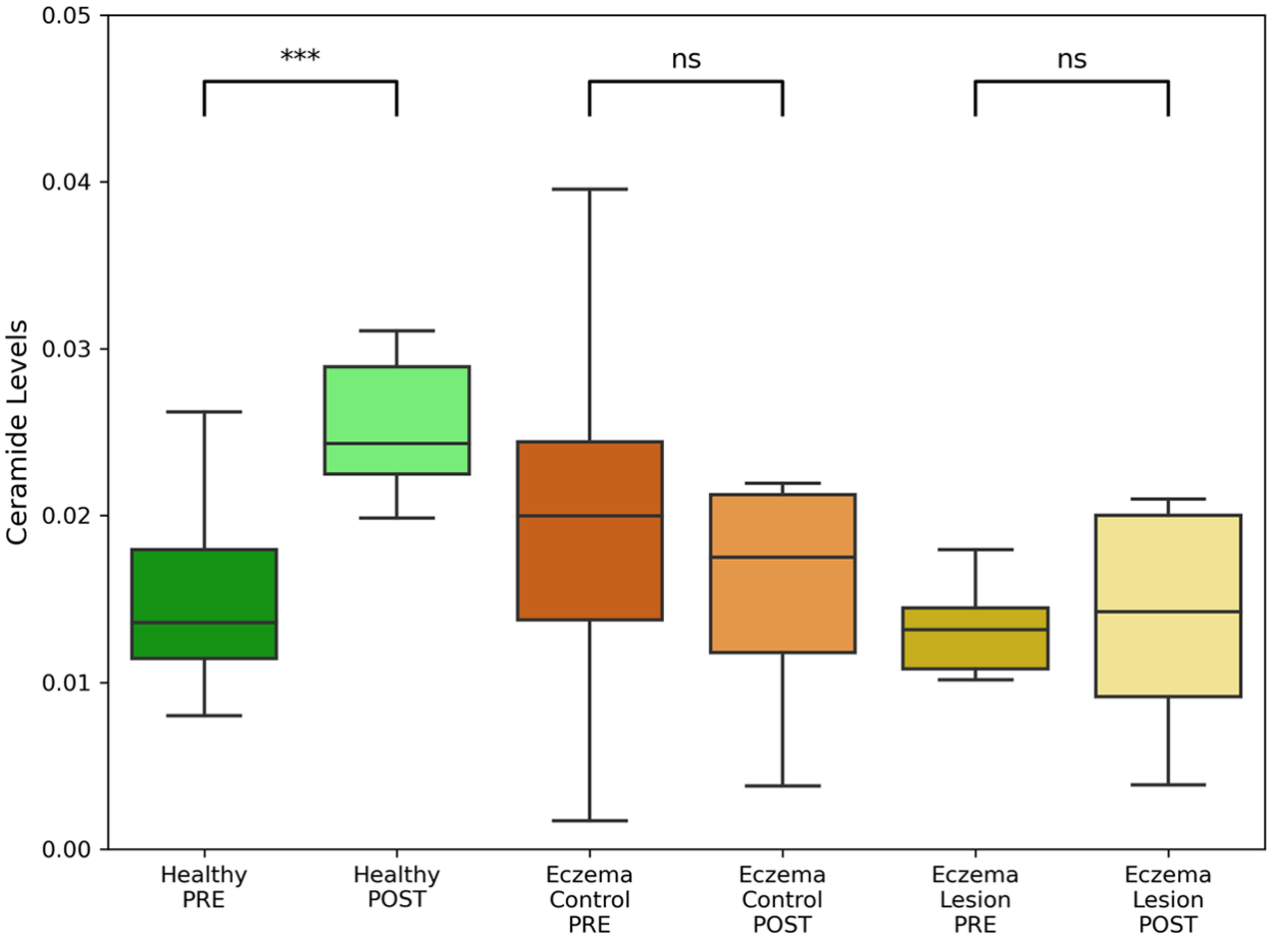
Ceramide content quantified from characteristic peaks (888 to $1436\;{\mathrm{cm}}^{-1}$) in deeper skin layers in healthy volunteers, eczema patients’ lesions or non-lesional (control) areas, pre-and post-application of test cream. ***p<0.001; n.s.: non-significant.

In the deeper layers, no increasing trend in ceramide levels was observed in either the eczema control or eczema lesion groups. However, in the healthy group, a three-star statistically significant increase in ceramide levels was noted post-application of the test cream as shown in Fig. 7.

## 4 Discussion

This exploratory clinical study aimed to assess the effects of a ceramide-based test cream on skin barrier function and hydration in both HV and patients with mild hand eczema (EP), by comparing single applications of the test cream and a control cream on skin physiological and biochemical endpoints.

The results indicate that although no statistically significant differences were observed in physiological parameters post-treatment, despite a trend of reduced TEWL and increased moisture content following test cream application, CRS-based water content measurements revealed a significant increase. This finding suggests that CRS is a more sensitive and reliable method for assessing skin hydration.

Although water has a relatively weak Raman scattering cross-section, its high abundance in skin produces a distinct O–H stretching band (3350 to $3550\;{\mathrm{cm}}^{-1}$) that can be quantitatively analysed using CRS. This approach has been validated in numerous *in vivo* studies,^1^^,^^4^ demonstrating consistent correlation between Raman-derived water profiles and hydration states measured by independent biophysical methods.

Other spectroscopic techniques, such as NIR spectroscopy, have also been widely used for noninvasive skin hydration assessment through the measurement of overtone and combination bands of water absorption.^29^^–^^33^ However, its spectral features are broad and nonspecific, often affected by overlapping signals from lipids and proteins.^34^^–^^36^ Moreover, NIR measurements represent an integrated signal from the bulk tissue and are not depth specific, providing only averaged information across the illuminated volume.^36^^,^^37^ By contrast, CRS offers superior molecular specificity and depth-resolved capability, enabling quantitative differentiation between water, ceramide, and keratin components within the SC.^3^^,^^23^^,^^38^^–^^42^ Although NIR can probe deeper layers due to lower optical scattering, CRS provides better spatial resolution and biochemical selectivity, making it more suitable for characterizing localized hydration dynamics and barrier function recovery following topical treatment.^35^^,^^36^^,^^43^ Overall, the results demonstrate that a single application of the test cream effectively enhanced skin barrier function in HV, whereas no such improvement was observed with the control cream.

Ceramide levels were quantified using a handheld CRS device, which enabled the evaluation of sphingoid-based fatty acids in both the superficial and deeper layers of the SC. This noninvasive assessment tool has been previously employed to quantify NMF levels in the skin of both AD patients and HV.^44^ A single application of either of the creams resulted in a significant increase in ceramide levels across all subjects, with a particularly greater increase observed following the application of the test cream. Notably, although the test cream significantly enhanced ceramide levels in both the superficial and deeper skin layers, the control cream only increased ceramide levels in the superficial layers of the SC. These findings suggest that the test cream may penetrate more deeply into the SC compared with the control cream.

In light of the evidence that ceramide levels tend to be lower in the skin of AD patients and the subsequent need to restore ceramide levels in these patients, ceramide-containing creams have gained increasing attention from dermatological investigators in recent years.^24^^,^^45^ In a prospective, open-label, observational, multicenter study by Koh et al., a ceramide-dominant therapeutic moisturizer (containing hydroxypropyl bispalmitamide MEA, as also contained in the test cream in the current study) administered twice daily for 6 weeks was shown to be safe and effective in the management of AD in young children.^46^ Our study further validated the positive effect of ceramide cream on the management of hand eczema.

Given this indication of deeper penetration and more efficient ceramide elevation, further analysis was conducted specifically on the side where the test cream was applied. The data from this side were categorized into three groups: healthy, eczema control, and eczema lesion. In the superficial layers, the test cream application led to an increase in ceramide levels across all three groups, with the healthy group demonstrating a greater statistically significant increase. Upon examining the deeper layers of the SC, the analysis revealed a significant increase in ceramide levels only in the healthy group, with no notable changes in the eczema control and eczema lesion groups.

The lack of increased ceramide levels in the deeper skin layers of the eczema control and eczema lesion groups post-application of the ceramide-based cream may be attributed to several factors. The study evaluated only a single application of the moisturizer, with measurements taken 2 h post-application, and the sample size was relatively small. Future studies could benefit from measuring ceramide levels at more frequent intervals, such as every 30 min. Given that EP often has a compromised skin barrier, it is possible that ceramide was absorbed but could not be retained within the skin due to the damaged barrier. Shorter measurement intervals could provide insight into whether ceramide was absorbed and subsequently lost from the SC. Moreover, the trend observed in the superficial layers of the EP, where ceramide levels increased but did not extend to the deeper layers, may indicate that the ceramide either did not penetrate deeply enough or, due to the compromised barrier, was lost before deeper penetration could occur. To address this, future studies should consider shorter measurement intervals and may incorporate a group using occlusion after ceramide application to evaluate its retention and penetration more effectively. In addition, repeated applications of the moisturizer over an extended period, as well as larger sample sizes, should be considered to further validate these findings.

## 5 Conclusion

This study successfully demonstrated the use of a dual-wavelength CRS system to analyze the biochemical components of the SC. The results underscore the promising potential of ceramide-based topical treatments in enhancing ceramide levels, thereby improving skin barrier function and hydration. The dual-wavelength CRS system proved to be a more sensitive and reliable tool for assessing skin hydration and barrier function, outperforming traditional methods such as TEWL and moisture content measurements. This highlights the CRS system’s capability to provide detailed, noninvasive insights into the skin’s biochemical composition and the effectiveness of topical treatments.

The results indicate that the test cream significantly increased ceramide levels in both the superficial and deeper layers of the SC, with greater penetration observed in comparison to the control cream. These findings provide strong support for the use of ceramide-enriched formulations in skincare. Although a single application of the test cream effectively improved skin hydration and barrier function in HV, the lack of similar improvements in the deeper layers of EP skin may be attributed to the compromised skin barrier typically seen in eczema.

These results highlight the need for further research, including more frequent measurement intervals, occlusion techniques, and repeated applications to better assess the retention and deeper penetration of ceramide-based treatments in patients with compromised skin barriers. Larger, more comprehensive studies are required to validate these preliminary findings and provide robust evidence of the long-term efficacy of ceramide-based treatments for managing skin conditions such as eczema.

## Supplementary Material

jbo-250264SSRR-s01.docx

10.1117/1.JBO.30.S3.S34117.s01

## Data Availability

Data are available upon request from the corresponding author.
