# Supplementary material for: Noninvasive assessment of skin barrier function: evaluating ceramide-based moisturizer using confocal Raman spectroscopy
Source: J Biomed Opt. 2025 Dec 31;30(Suppl 3):S34117. doi: 10.1117/1.JBO.30.S3.S34117 (PMC12770911; doi:10.1117/1.JBO.30.S3.S34117)
Supplement: Supplementary file 1 [file JBO_030_S34117_SD001.pdf]

## Supplementary materials

The skin of patients with AD is characterized by elevated skin-surface-pH and abnormal barrier function that prognosticates the course of the condition. Skin surface pH has been found to increase in both lesional and non-lesional sites in AD. This elevation in pH contributes to the activation of pro-inflammatory cytokines and a reduction in the production of antimicrobial peptides, thereby compromising the skin's innate antimicrobial defence. Given these implications, monitoring and therapeutic modulation of skin surface pH are essential components in the management of AD. In the present study, the ceramide-containing test cream—Ceradan® Advanced Hand Balm (Hyphens Pharma, Singapore)—was formulated to maintain a pH level comparable to that of healthy skin. Comprehensive measurements of skin pH were performed to evaluate the effectiveness of both creams.

At baseline, skin surface pH was significantly higher in EPs' lesions compared with HV skin ($p$=0.010). The test cream significantly reduced skin pH in HV and EP lesions (both $p$<0.0001). Similarly, control cream reduced skin pH in both groups but with less statistical significance ($p$=0.003 and 0.004, respectively). The magnitude of the reduction in mean pH was greater for test cream *vs* control cream in both HV (0.64 *vs* 0.26) and EP (0.70 *vs* 0.18). Therefore, the ability of a test cream to lower skin pH by a larger magnitude is important especially for patients with eczematous lesions. These results illustrate the benefit of the test cream which was specifically formulated to have a skin pH-lowering effect.

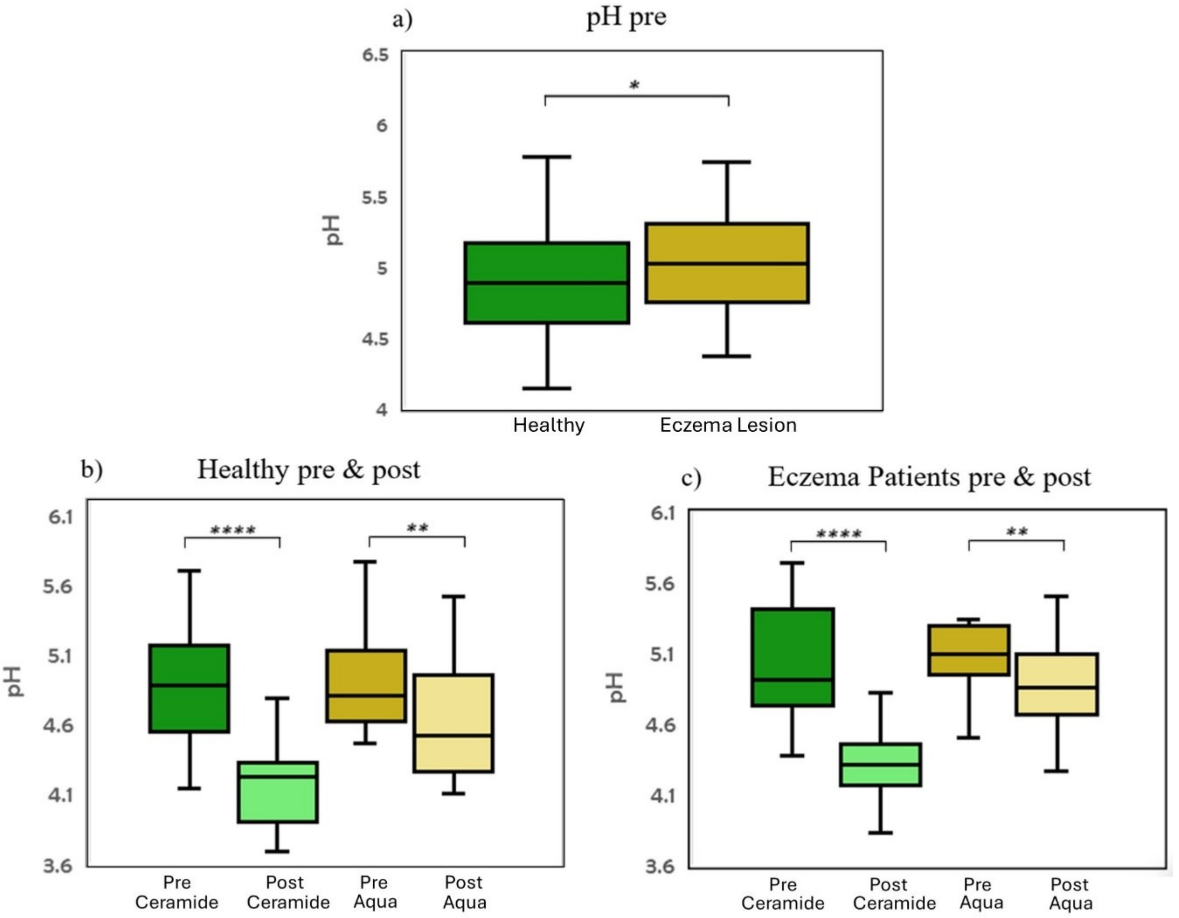


**Figure S1** Box plots of pH values showing the difference between pre- and post-application of ceramide cream and aqueous (****: $p<0.0001$; **: $p < 0.01$; *: $p < 0.05$;) (a) pH of HV and EP prior to cream application, (b) pH of HV pre and post treatment, and (c) pH of EP pre and post treatment
